# Twin-Tool Orientation Synchronous Smoothing Algorithm of Pinch Milling in Nine-Axis Machine Tools

**DOI:** 10.3390/ma17122977

**Published:** 2024-06-18

**Authors:** Dongdong Song, Shuai Zhu, Fei Xue, Yagang Feng, Bingheng Lu

**Affiliations:** 1 College of Mechanical and Electronic Engineering, Northwest A&F University, No.3 Taicheng Road, Yangling District, Xianyang 712100, China; zs1124@nwafu.edu.cn; 2 State Key Laboratory for Manufacturing Systems Engineering, Xi’an Jiaotong University, West Building 5, No. 99 Yan Cheung Road, Yanta District, Xi’an 710054, China; bhlu@mail.xjtu.edu.cn; 3 Xi’an Aerospace Huawei Chemical & Biotechnology Co., Ltd., No. 289 Feitian Road, Aerospace Industry Park, Chang’an District, Xi’an 710054, China; fyg315@126.com

**Keywords:** pinch milling, twin-tool orientation, smoothing orientation planning method, feasible domain

## Abstract

Pinch milling is a new technique for slender and long blade machining, which can simultaneously improve the machining quality and efficiency. However, two-cutter orientation planning is a major challenge due to the irregular blade surfaces and the structural constraints of nine-axis machine tools. In this paper, a method of twin-tool smoothing orientation determination is proposed for a thin-walled blade with pinch milling. Considering the processing status of the two cutters and workpiece, the feasible domain of the twin-tool axis vector and its characterization method are defined. At the same time, an evaluation algorithm of global and local optimization is proposed, and a smoothing algorithm is explored within the feasible domain along the two tool paths. Finally, a set of smoothly aligned tool orientations are generated, and the overall smoothness is nearly globally optimized. A preliminary simulation verification of the proposed algorithm is conducted on a turbine blade model and the planning tool orientation is found to be stable, smooth, and well formed, which avoids collision interference and ultimately improves the machining accuracy of the blade with difficult-to-machine materials.

## 1. Introduction

Pinch milling uses two cutters that can simultaneously mill the opposing surfaces of two blades from top to top, which can significantly improve machining efficiency and quality. Turbine blades are typically elongated components, distinguished by thin walls, asymmetry, and twisted free surfaces. A twin-tool orientation, formed by three rotary axes, plays a crucial role in machining complex parts like a turbine blade. Each tool orientation requires continuous and smooth transitions, but it is very difficult to simultaneously plan these tool orientations. Meanwhile, inappropriately defined tool orientations in such applications can cause fatal collisions and damage the in-processing part, and unsmooth segments of tool orientation may lead to unwanted vibrations due to sudden fluctuations in tool movements and, thus, limit the full production capabilities of multiaxis machine tools and inevitably impact machining precision.

In order to obtain smooth tool orientation, many studies have been conducted to achieve optimal tool orientation. For example, the tool-sweep surface-based method [1], curvature matching method [2,3,4], multipoint machining method [5,6,7], and simultaneous optimization with feed direction [8,9]; all share the goal of selecting a noninterference and collision-free tool orientation. Meanwhile, Mi et al. [10] presented a feasible C-space computation algorithm for triangular mesh models to plan smooth tool orientation along a tool path. Chen et al. [11] presented a reference plane to generate a set of smoothly aligned tool orientations along a tool path. Gong et al. [12] proposed a method to find the optimal tool orientation based on a ruled surface; the optimization objective function was set to minimize the vibration of rotary axes. Hong et al. [13] described a practical method for tool orientation generation for ball cutters commonly used in complex-surface-finish machining. Dong et al. [14] proposed a multiscale tool orientation generation method that considers both the machining strip width and roughness scales. Sun et al. [15] presented the prediction of an automatic tool axis orientation algorithm that avoids chatter while improving productivity.

In recent years, researchers’ attention has also been directed to smoothing tool orientation, and researchers have proposed many typical methods, such as the shortest-path linking method [16], interpolation method [17,18], and optimization-based method [19,20,21]. Yuan et al. [22] proposed a method for generating smooth tool orientation by considering the relationship between tool orientation and strip width for five-axis machining with flat-end cutters. Dong et al. [23] proposed a multiscale tool orientation smoothness method that considers both machining strip width and roughness scales. On this basis, a novel method based on the best curvature matching was used to generate smoothing tool orientation [24]. Yana et al. [25] proposed a mathematical framework to generate smoothing tool axis variation even on partial surfaces lacking G2 and/or G1 continuities. Wang et al. [26] constructed a selection strategy for the smoothing tool axis from the discrete domain of feasible orientations. All the technology deeply improved the smoothness of tool orientation vector sequences.

The above theoretical studies are dedicated to planning smoothing tool axis vectors in single-tool machining. But pinch milling has two cutters, and any nonsmooth tool axis direction can seriously affect the cutting process. In early stages, the twin-tool milling path is planned [27], the opposite cutting contact points and paths are planned, the twin-tool orientation is characterized, and the initial dual-tool axis vectors are also planned. However, in actual machining, it was found that the angle between the cutters changed discontinuously and unevenly, which limited the cutting speed of the machine tool and caused obvious vibration during cutting processes. Meanwhile, the envelope surface is jointly constructed by the tool and workpiece at the cutting contact, which plays a crucial role in the cutting process. The state of the tool orientation has a significant impact on the formation of the envelope surface and the machining quality of the workpiece surface. It is a key item in the twin-tool cutting process. Therefore, smoothness orientation planning for pinch milling is an enormous challenge.

In this paper, the twin-tool orientation synchronous smoothing algorithm is proposed for thin-wall blades used in pinch milling. Considering the two cutters’ structural layouts, the twin-tool orientations and coupling relationship are characterized; a method is also proposed for defining the feasible region of the cutters. In order to guarantee the smoothness of the tool orientations, the tool axis vector is parameterized and tool posture curve of global optimization is formulized, and the evaluation algorithm of local optimization is investigated. Finally, the twin-tool orientation selection and optimization scheme is explored within the feasible domain along the two tool paths. Furthermore, the smoothness tool orientation is planned for a typical turbine blade.

## 2. Twin-Tool Orientations Identification

### 2.1. The Method of Pinch Milling

**Figure 1:**
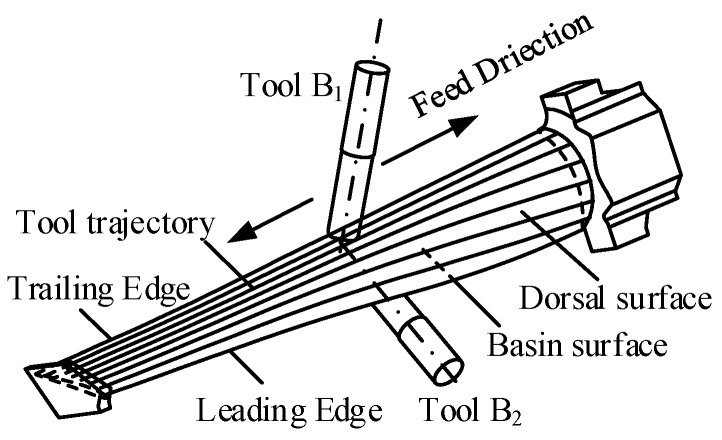
The method of pinch milling.

Pinch milling is a method that involves simultaneously milling both sides of an irregular blade profile from top to top, using two tools concurrently. The milling process is shown in Figure 1; two tools are oppositely and simultaneously assigned to mill the dorsal (convex) and basin (concave) surfaces, and the directions along blade height are the feed directions for the two tools. Meanwhile, it considers the shape and size characteristics of the leading and trailing edges in order to avoid collisions between the two cutters, which are milled along the length direction with either one of the twin tools.

At the top-to-top contact point, the tool axes orientations of the two cutters should influence each other, and the rotation angle of the workpiece simultaneously affects the cutting angle of the tool.

### 2.2. Twin-Tool Orientations Description

**Figure 2:**
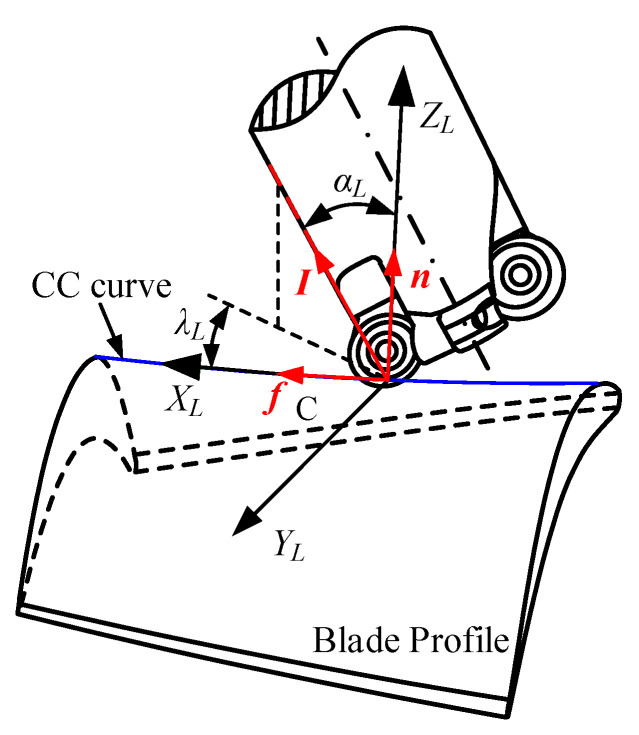
Definition of tool orientation angles.

In pinch milling, in addition to the translations along the length direction of the blade, two cutters can be rotated with the workpiece axis to adapt to complex curved surfaces. As shown in Figure 2, the local coordinate system *O_L_*–*X_L_*–*Y_L_*–*Z_L_* is defined at the cutter contact (CC) point C. The *X_L_*-axis is defined along the instantaneous cutting direction, the *Z_L_*-axis is defined along the direction of local surface normal, and the *Y_L_*-axis is defined by the *X_L_*- and *Z_L_*-axes with the right-hand-rule. The tool orientation is determined with an inclination angle *α_L_* around the *Y_L_*-axis and a tilt angle *λ_L_* around the *Z_L_*-axis. The tool orientation for the other tool is also determined by the same rule.

In the local coordinate system *O_L_*–*X_L_*–*Y_L_*–*Z_L_*, the unit vectors ***f***, ***n***, and ***I*** are defined along the direction of the *X_L_*-axis, *Z_L_*-axis, and tool-axis, respectively. Then, the inclination angle *α_L_* and the tilt angle *λ_L_* can be derived as follows:

(1) $$\begin{array}{l} \alpha_{L}=\cos^{-1}(n\cdot I)=\cos^{-1}(xx_{n}+yy_{n}+zz_{n})\\ \lambda_{L}=\cos^{-1}(\frac{I\cdot f}{\sin\alpha_{L}})=\cos^{-1}(\frac{xx_{f}+yy_{f}+zz_{f}}{\sin\alpha_{L}}) \end{array}$$

where $f=[x_{f},y_{f},z_{f}]$, $n=[x_{n},y_{n},z_{n}]$, I=[x,y,z].

**Figure 3:**
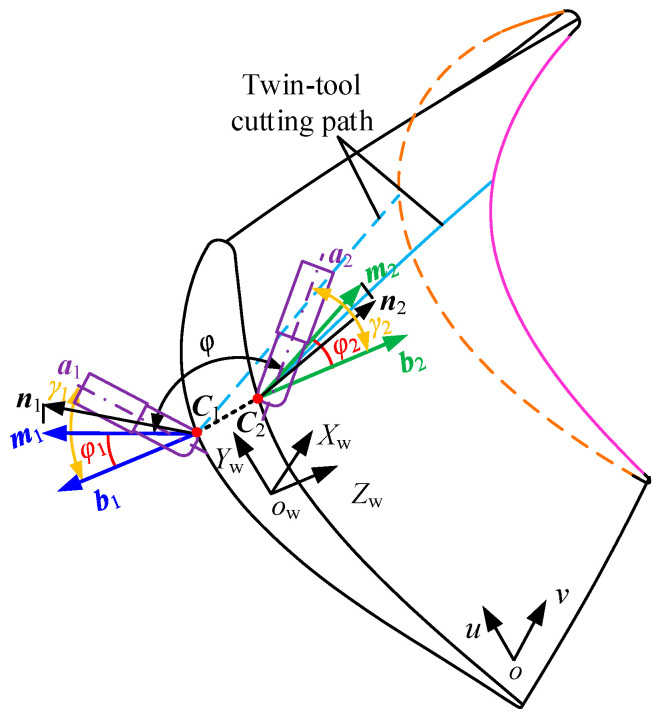
Twin-tool orientations constraint relationship of the CC point.

Considering structural constraints of pinch milling, the two tools can only horizontally swing and parallel each other. As Figure 3, in the workpiece coordinate system *O_W_*–*X_W_*–*Y_W_*–*Z_W_*, the points C_1_ and C_2_ are top-to-top cutting contacts, ***a*_1_** and ***a*_2_** are supposed to be the tool orientations at the opposite cutting points, respectively, ***n*_1_** and ***n*_2_** are supposed to be the normal vectors at the point on the dorsal and basin surfaces respectively, then ***m*_1_** and ***m*_2_** are the projection vectors of ***n*_1_** and ***n*_2_** onto the plane *Y_W_*–*O_W_*–*Z_W_*.

Assuming normal vector ***n*_1_** is [*x*_1_, *y*_1_, *z*_1_] and normal vector ***n*_2_** is [*x*_2_, *y*_2_, *z*_2_], then [0, *y*_1_, *z*_1_] is expressed as the vectors ***m*_1,_** and [0, *y*_2_, *z*_2_] is expressed as the vectors ***m*_2_**. The angle between the vectors ***m*_1_** and ***m*_2_** is denoted as *φ*, which can be expressed as

(2) $$\varphi =\cos^{-1}(\frac{y_{1}y_{2}+z_{1}z_{2}}{\sqrt{{y_{1}}^{2}+{z_{1}}^{2}}\sqrt{{y_{2}}^{2}+{z_{2}}^{2}}})$$

The vectors ***b*_1_** and ***b*_2_** are the projection vectors of ***a*_1_** and ***a*_2_** onto the plane *Y_W_*–*O_W_*–*Z_W_*, respectively. Suppose that the angle between ***m*_1_** and ***b*_1_** is *φ*_1_, and the angle between ***m*_2_** and ***b*_2_** is ***φ*_2_**, then the relationship among *φ*_1_, *φ,* and *φ*_2_ can be expressed as follows:

(3) $$\varphi_{1}+\varphi +\varphi_{2}=\pi$$

Suppose that the angle between ***a*_1_** and ***b*_1_** is *γ*_1_, which is the rotation angle from ***a*_1_** to ***b*_1_**; Similarly, the angle between ***a*_2_** and ***b*_2_** is *γ*_2_, which is the rotation angle from ***a*_2_** to ***b*_2_**. Then translating the vectors into the same plane *X_W_*–*O_W_*–*Z_W_*, ***a*_1_** and ***a*_2_** can be calculated and expressed as

(4) $$\begin{array}{l} a_{1}=(\sin\gamma_{1}(y_{n1}\sin\varphi_{1}+z_{n1}\cos\varphi_{1}),y_{n1}\cos\varphi_{1}-z_{n1}\sin\varphi_{1\;},\\ \;\;\;\;\;\;\;\;\;\;\cos\gamma_{1}(y_{n1}\sin\varphi_{1}+z_{n1}\cos\varphi_{1}))\\ a_{2}=(\sin\gamma_{2}(y_{n2}\sin(\varphi +\varphi_{1})+z_{n2}\cos(\varphi +\varphi_{1})),-y_{n2}\cos(\varphi +\varphi_{1})+z_{n2}\sin(\varphi +\varphi_{1}),\\ \;\;\;\;\;\;\;\;\;\;-\cos\gamma_{2}(y_{n2}\sin(\varphi +\varphi_{1})+z_{n2}\cos(\varphi +\varphi_{1}))) \end{array}$$

where ***n***_1_ = [*x*_n1_, *y*_n1_, *z*_n1_], and ***n***_2_ = [*x*_n2_, *y*_n2_, *z*_n2_].

### 2.3. Twin-Tool Orientations Coupling Relationship

Local coordinate systems *O*_L1_*X*_L1_*Y*_L1_*Z*_L1_ and *O*_L2_*X*_L2_*Y*_L2_*Z*_L2_ are established at the point C_1_ and C_2_ according to the right-hand rule; ***f*_1_** is the unit vector of tool feed direction at the point C_1_, and ***f*_2_** is the unit vector of the tool feed direction at the point C_2_. If the tool axis vector ***a*_1_** and ***a*_2_** are all unit vectors, the inclination angle *α*_L1_ and tilt angle *λ*_L1_ of the cutter at the dorsal surface can be derived as follows:

(5) $$\alpha_{L1}=\cos^{-1}(\frac{\begin{array}{l} \sin\gamma_{1}(y_{n1}\sin\varphi_{1}+z_{n1}\cos\varphi_{1})x_{n1}+(y_{n1}\cos\varphi_{1}-z_{n1}\sin\varphi_{1\;})y_{n1}\\ +z_{n1}\cos\gamma_{1}(y_{n1}\sin\varphi_{1}+z_{n1}\cos\varphi_{1}) \end{array}}{\sqrt{{\left(y_{n1}\right)}^{2}+{\left(z_{n1}\right)}^{2}}})$$

(6) $$\lambda_{L1}=\cos^{-1}(\frac{\begin{array}{l} \sin\gamma_{1}(y_{n1}\sin\varphi_{1}+z_{n1}\cos\varphi_{1})x_{f1}+(y_{n1}\cos\varphi_{1}-z_{n1}\sin\varphi_{1\;})y_{f1}\\ +\cos\gamma_{1}(y_{n1}\sin\varphi_{1}+z_{n1}\cos\varphi_{1})z_{f1} \end{array}}{\sin\alpha_{L1}\sqrt{{\left(y_{n1}\right)}^{2}+{\left(z_{n1}\right)}^{2}}})$$

where ***f***_1_ = [*x*_f1_, *y*_f1_, *z*_f1_].

Similarly, the inclination angle *α*_L2_ and tilt angle *λ*_L2_ of the cutter at the basin surface can be derived as follows:

(7) $$\alpha_{L2}=\cos^{-1}(\frac{\begin{array}{l} \sin\gamma_{2}(y_{n2}\sin(\varphi +\varphi_{1})+z_{n2}\cos(\varphi +\varphi_{1}))x_{n2}+(-y_{n2}\cos(\varphi +\varphi_{1})+z_{n2}\sin(\varphi +\varphi_{1}))y_{n2}\\ +(-\cos\gamma_{2}(y_{n2}\sin(\varphi +\varphi_{1})+z_{n2}\cos(\varphi +\varphi_{1})))z_{n2} \end{array}}{\sqrt{{\left(y_{n2}\right)}^{2}+{\left(z_{n2}\right)}^{2}}})$$

(8) $$\lambda_{L2}=\cos^{-1}(\frac{\begin{array}{l} \sin\gamma_{2}(y_{n2}\sin(\varphi +\varphi_{1})+z_{n2}\cos(\varphi +\varphi_{1}))x_{f2}+(-y_{n2}\cos(\varphi +\varphi_{1})+z_{n2}\sin(\varphi +\varphi_{1}))y_{f2}\\ +(-\cos\gamma_{2}(y_{n2}\sin(\varphi +\varphi_{1})+z_{n2}\cos(\varphi +\varphi_{1})))z_{f2} \end{array}}{\sin\alpha_{L2}\sqrt{{\left(y_{n2}\right)}^{2}+{\left(z_{n2}\right)}^{2}}})$$

where ***f***_2_ = [*x*_f2_, *y*_f2_, *z*_f2_].

It will be seen that the rotation angle *φ*_1_ simultaneously determines the inclination angles *α*_L1_ and *α*_L2_, as well as the tilt angles *λ*_L1_ and *λ*_L2_ of the tool axis vectors, resulting in mutual influence between the two tool axis vectors without a linear relationship. Meanwhile, the sizes of *α*_L1_ and *λ*_L1_ of the tool orientation ***a*_1_** are directly affected by the parameter of the angle *γ*_1_, and the sizes of *α*_L2_ and *λ*_L2_ of the tool orientation ***a*_2_** are directly affected by the parameter of the angle *γ*_2_. This phenomenon indicates that there is a mutually dependent coupling relationship between the two tool axis vectors, meaning that when the parameters of one tool axis vector change, it triggers corresponding changes in the parameters of the other tool axis vector. This interrelated state directly influences the machining conditions during the twin-tool cutting process. Therefore, adjustments to the parameters of either tool axis vector require careful consideration of their impact on the overall machining state.

## 3. Tool Orientation Smoothing Optimization Formulation

### 3.1. Identification of Twin-Tool Orientation Accessible Region

Due to the structural constraints of the pinch milling, the two tool movement spaces are all imposed limitations, and we thoroughly delved into the mutual influence relationship. Based on the parameters of *φ*_1_, *γ*_1_, and *γ*_2_, we can solve the preliminary feasible space of all tool axis vectors, which all satisfy the structural constraints. This specific space is defined as the feasible domain of *Q*(*α*_L1_, *λ*_L1_, *α*_L2_, *λ*_L2_) and can be specifically described as

(9) $$Q(\alpha_{L1},\lambda_{L1},\alpha_{L2},\lambda_{L2})=\left\{a_{1},\left.a_{2}\right|\;\varphi_{1},\gamma_{1},\gamma_{2}\right\}$$

Under the premise of satisfying the structural constraints on pinch milling, due to multiple factors such as tool size, blade, and fixture shapes, the tool axis vector cannot cover all ranges within the feasible domain *Q*. In addition, more constraints are considered to avoid collisions and interferences. On the top-to-top cutting contact, the parameters are calculated based on tool data and local geometric data of the curved surface, such as the distance *d*_e_ between the tool and the machine tool or workpiece, the effective cutting radius *R*_e_ of the tool, the radius of curvature *ρ*_k_ at the contact point, and the distance *d*_s_ between the tool bottom and the workpiece. Using these data, we can determine a collision-free or interference-free tool axis vector space. In the twin-tool milling process, any of these tools must be ensured to avoid collisions and interferences. Therefore, the feasible domain is further restricted and marked as *R*_1_(*α*_L1_, *λ*_L1_, *α*_L2_, *λ*_L2_) and can be specifically described as

(10) $$R_{1}(\alpha_{L1},\lambda_{L1},\alpha_{L2},\lambda_{L2})=\left\{a_{1},\left.a_{2}\right|\;d_{e}>0,R_{e}<\rho_{k},d_{s}>0\right\}$$

Meanwhile, the geometry of the cutting surface is affected by the angle of the tool orientation, which manifests as excessive residual heights, as well as overcutting and undercutting phenomena, thereby affecting the machining quality of the workpiece surface. Thus, under the condition of the allowable scallop height *h*_max_ and chord error *δ*_tmax_ requirements, the reachable motion space of the tool axis vector can be determined, which is defined as the feasible region *R*_2_(*α*_L1_, *λ*_L1_, *α*_L2_, *λ*_L2_) under geometric error constraints, and is expressed as

(11) $$R_{2}(\alpha_{L1},\lambda_{L1},\alpha_{L2},\lambda_{L2})=\left\{a_{1},\left.a_{2}\right|\;h_{r}<h_{rmax},\delta_{tr}<\delta_{tmax}\right\}$$

where *h*_r_ is the actual scallop height and *δ*_tr_ is the actual chord error corresponding to the two-tool axis vectors.

**Figure 4:**
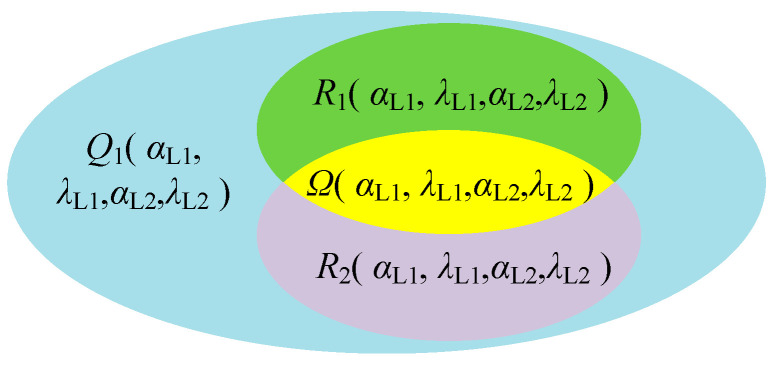
Twin-tool orientations feasible region.

The true feasible region is actually the intersection of all feasible regions considered under the constraints, thus forming a closed area reachable by the twin-tool axis vectors, as shown in Figure 4. This area is defined as the feasible region *Ω*(*α*_L1_, *λ*_L1_, *α*_L2_, *λ*_L2_) for the posture of dual tools, and is represented as

(12) $$\Omega (\alpha_{L1},\lambda_{L1},\alpha_{L2},\lambda_{L2})=Q\cap R_{1}\cap R_{2}$$

Assuming that the feasible region of ***a***_1_ is *Ω*_1_(*α*_L1_, *λ*_L1_), and the feasible region of ***a***_2_ is *Ω*_2_(*α*_L2_, *λ*_L2_), the final feasible region is the union of the feasible regions of the two tool axis vectors. The equivalent expression *Ω* is represented as

(13) $$\Omega (\alpha_{L1},\lambda_{L1},\alpha_{L2},\lambda_{L2})=\Omega_{1}(\alpha_{L1},\lambda_{L1})\cup \Omega_{2}(\alpha_{L2},\lambda_{L2})$$

**Figure 5:**
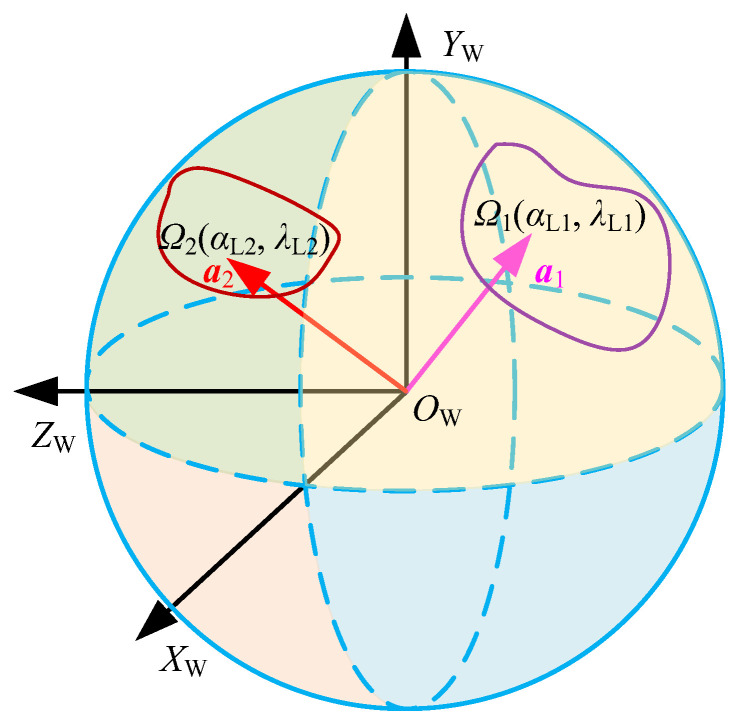
Feasible region in the workpiece coordinate system.

In addition, to clearly describe the positions of the dual tool axis vectors in the workpiece coordinate system *O*_W_*X*_W_*Y*_W_*Z*_W_, a Gaussian sphere of unit tool axis vectors is constructed at the origin *O*_W_. Meanwhile, the unit vector ***a***_1_ and ***a***_2_ are translated to the origin *O*_W_, respectively, so that ***a***_1_ and ***a***_2_ are equivalent to a particle on the Gaussian sphere, and the corresponding feasible regions *Ω*_1_ and *Ω*_2_ represent the ranges of motion of the particles on the Gaussian sphere surface, as shown in Figure 5. However, the inclination angle *α_L_* and tilt angle *λ_L_* only describe the tool’s posture in the local coordinate system at the contact point, and its tool axis vector is actually a directional vector in the workpiece coordinate system, which can be represented as (*i*, *j*, *k*). Therefore, the feasible region *Ω* of twin-tool orientation can be re-expressed as

(14) $$\Omega (i_{W1},j_{W1},k_{W1},i_{W2},j_{W2},k_{W2})↔\Omega (\alpha_{L1},\lambda_{L1},\alpha_{L2},\lambda_{L2})$$

### 3.2. Global Smoothness of Twin-Tool Orientation

**Figure 6:**
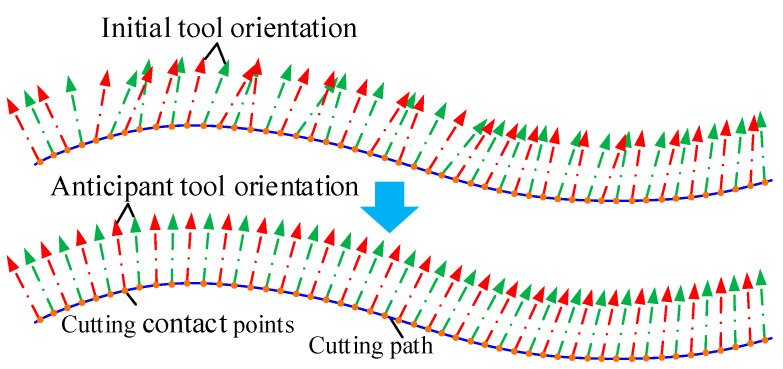
Global smoothness of tool orientation.

In multiaxis machining, it is expected that the tool axis vectors change continuously and uniformly along the contact points to achieve global smoothing of the tool axis vectors. However, the tool axis vectors formed during trajectory planning may have excessive or uneven angular changes, as shown in Figure 6, and the concept of “smoothness” is inherently complicated and often inconsistent. For unit tool axis vectors, rotation angle of tool orientation is equivalent to the trajectory of a particle moving on the spherical surface. The smoothing is transformed into a curve fitting problem at the particle, which means finding a smooth curve that ensures all tool axis vectors lie on this curve.

Assuming the tool axis vector at the cutting contact point is defined as ***a***_c*k*_, and the values are [*a_i_*,_c*k*_, *a_j,_*_c*k*_, *a_k_*,_c*k*_], where the subscripts *i*, *j*, *k* represent the component values in each axis of the coordinate system, and the subscript c*k* indicates the position of the cutting contact point), and the corresponding control vertex is ***a****_i_*, then the parametric B-spline curve can be defined by the basis functions *N_i,q_*(*s*), control points ***a****_i_*, and degree *n* with the following form:

(15) $$P_{c}(s)=\sum_{i=0}^{n}N_{i,q}(s)a_{i}$$

The basis functions *N_i,q_*(*s*) are functions of the geometric parameter *s* and knot vector *S* = [*s*_0_, *s*_1_, …, *s*_n+1_], and are defined as follows:

(16) $$\begin{array}{l} N_{i,0}(s)=\left\{\begin{array}{l} 1,\;\;\;\;\;\;\;\;s\in \left[s_{i},s_{i+1}\right]\\ 0,\;\;\;\;\;\;\;\;s\notin \left[s_{i},s_{i+1}\right] \end{array}\right.\;\;\;\;\;\;\;\;s\in \left[0,1\right]\\ N_{i,q}(s)=\frac{s-s_{i}}{s_{i+p}-s_{i}}N_{i,q-1}(s)+\frac{s_{i+p+1}-s}{s_{i+p+1}-s_{i+1}}N_{i+1,q-1}(s),\;\;q\geq 1 \end{array}$$

Meanwhile, in order to achieve the analytical solution of control points, the number of basis functions and control points in Equation (14) is set equal to the number of tool axis vector *a_k_*, which allows for the linear system to be solved for the control points. For this, the knot vector *S* is solved based upon the angles between the tool axis vector ***a****_k_*. Thus, the parameter values ${\bar{s}}_{k}$ are assigned to each tool axis vector ***a****_k_*, which can be expressed as

(17) $$\left\{\begin{array}{l} d=\sum_{k=1}^{n}\sqrt{\cos^{-1}(a_{k}\cdot a_{k-1})}\\ {\bar{s}}_{0}=0,\;{\bar{s}}_{n}=1\\ {\bar{s}}_{k}={\bar{s}}_{k-1}+\frac{\sqrt{\cos^{-1}(a_{k}\cdot a_{k-1})}}{d},k=1,\cdots ,n-1 \end{array}\right.$$

Using the assigned parameter values ${\bar{s}}_{k}$, the knot vector is solved as follows:

(18) $$\left\{\begin{array}{l} s_{0}=\cdots =s_{p}=0,\;s_{n+1}=\cdots =s_{n+q+1}=1\\ s_{j+n}=\frac{1}{q}\sum_{k=j}^{j+n-1}{\bar{s}}_{k},j=1,\cdots ,n-q \end{array}\right.$$

Based on the defined knot vector S and the assigned parameter values ${\bar{s}}_{k}$, the control point ***a****_i_* can be solved through the line system of equations and represented as

(19) $$\begin{array}{l} \underset{\Phi }{\underset{︸}{\left[\begin{array}{ccc} N_{0,q}({\bar{s}}_{0}) & \cdots & N_{n,q}({\bar{s}}_{0})\\ \vdots & \ddots & \vdots \\ N_{0,q}({\bar{s}}_{n}) & \cdots & N_{n,q}({\bar{s}}_{n}) \end{array}\right]}}\underset{\Gamma }{\underset{︸}{\left[\begin{array}{c} {a_{0}}^{T}\\ \vdots \\ {a_{n}}^{T} \end{array}\right]}}=\underset{\Upsilon }{\underset{︸}{\left[\begin{array}{c} {a_{c0}}^{T}\\ \vdots \\ {a_{cn}}^{T} \end{array}\right]}}\\ \;\Gamma =\Phi^{-1}\Upsilon \end{array}$$

Once the knot vector S and control points ***a****_i_* have been determined, the B-spline curve *P*_c_(s) of the tool axis can be obtained from Equation (14), which is called the tool posture curve *P*_c_(s), and describes the trajectory of a particle on the spherical surface, representing the angle variation of the tool axis vector at the contact point. Therefore, new tool axis vectors are interpolated the contact point along the tool path; all tool axis vectors are located on the tool posture curve, and the global smoothing tool axis vector can be obtained.

### 3.3. Local Smoothness of Twin-Tool Orientation

All cutting contact points are all expected to have tool axis vectors that achieve global smoothness variation. However, the optimized cutting axis vector may not be in the feasible region, resulting in the uncertainty of the local cutting contact, so it is necessary to further solve the local optimization problem of the cutting axis vector. For the discrete data, the variation of the tool axis vector can be described as local angular velocity, which can be approximately represented by mean angular velocity in each interval between two neighboring tool orientations. Assuming that the tool center has a constant feed rate, the average angular velocity at unit speed of the tool axis vector between the *i*th interval can be expressed as

(20) $$\omega_{i}=\frac{\theta_{i}}{l(c_{i}c_{i+1})}$$

where *θ_i_* is the angle between tool axis vectors ***a****_i_* and ***a****_i+_*_1_, and $l(c_{i}c_{i+1})$ stands for the arc length between the two cutting contact points.

In real situations, usually, two consecutive cutting contact points are close, and *θ_i_* is small, so the approximate relationship between angles and arc length can be expressed as

(21) $$\begin{array}{l} l(c_{i}c_{i+1})\approx \left\|c_{i}c_{i+1}\right\|\\ \theta_{i}\approx \sin\theta_{i}\approx \left\|a_{i+1}-a_{i}\right\| \end{array}$$

Therefore, the angular speed *ω_i_* are re-expressed as

(22) $$\omega_{i}=\frac{\left\|a_{i+1}-a_{i}\right\|}{\left\|c_{i}c_{i+1}\right\|}$$

The smoothness of angular velocity can be achieved by minimizing the harmonic mean of *ω_i_* or the almost constant *ω_i_* along the tool path to ensure the optimal tool axis vector. Then, the objective function for the optimization problem can be formulated as

(23) $$\begin{array}{l} H=\underset{\left\{a_{1},a_{2},\cdots ,a_{n}\right\}}{\min}\sum_{i=1}^{n-1}{\omega_{i}}^{2}\\ \;s.t.\;\alpha_{i},\beta_{i}\in \Omega \end{array}$$

The optimal tool axis vector is sought to achieve uniform and smooth variation of the tool axis at adjacent consecutive contact points within the feasible region.

### 3.4. Twin-Tool Orientation Selection and Optimization Schemes

**Figure 7:**
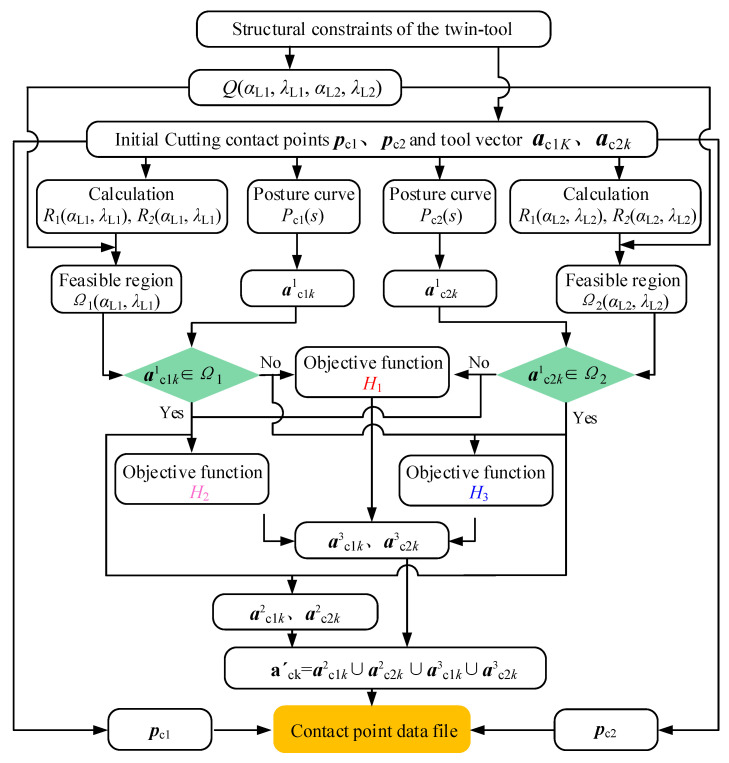
Flowchart of twin-tool orientation for pinch milling.

As long as the optimization algorithm is clearly defined, the tool posture determination process can be carried out. For the pinch milling, both the orientation of two tools are required to have continuous and smooth changes along the cutting path. Therefore, the selection and optimization process is established to solve the optimal tool axis vector; the flowchart of twin-tool smoothness orientation planning is shown in Figure 7.

For the two cutters on the dorsal and basin surfaces of the blade, all reachable regions *Q*(*α*_L1_, *λ*_L1_, *α*_L2_, *λ*_L2_) of the twin-tool axis vector are determined based on the structural constraints. And the feasible domain *Ω*(*α*_L1_, *λ*_L1_, *α*_L2_, *λ*_L2_) is calculated based on the cutting contact points ***p***_c1_ and ***p***_c2_ on the cutting path and the tool axis vectors ***a***_c1*k*_ and ***a***_c2*k*_. At the same time, the tool posture curves *P*_c1_(*s*) and *P*_c2_(*s*) are determined corresponding to the primitive tool axis, and the global optimal vectors ***a***^1^_c1*k*_ and ***a***^1^_c2*k*_ are solved. If ***a***^1^_c1*k*_ and ***a***^1^_c2*k*_ both belong to *Ω*, simultaneous optimization vectors on both sides can be achieved, which can be expressed as ***a***^2^_c1*k*_ and ***a***^2^_c2*k*_. However, it is difficult to achieve simultaneous optimization in the actual calculation process. For ***a***^1^_c1*k*_ and ***a***^1^_c2*k*_, which do not belong to *Ω*, it is necessary to ensure that the change in angular velocity for each of the cutters is minimized. The objective function *H*_1_ is represented as

(24) $$\begin{array}{l} H_{1}=\underset{\left\{a_{c11},a_{c21},\cdots ,a_{c1n},a_{c2n}\right\}}{\min}\sum_{i=1}^{n-1}({\omega_{c1i}}^{2}+{\omega_{c2i}}^{2})\\ \;s.t.\;a_{c1k}^{1}\notin \Omega ,\;a_{c2k}^{1}\notin \Omega \end{array}$$

If ***a***^1^_c1*k*_ belongs to *Ω* and ***a***^1^_c2*k*_ does not belong to *Ω*, then the objective function *H*_2_ is represented as

(25) $$\begin{array}{l} H_{2}=\underset{\left\{a_{c11},a_{c21},\cdots ,a_{c1n},a_{c2n}\right\}}{\min}\sum_{i=1}^{n-1}{\omega_{c1i}}^{2}\\ \;s.t.\;a_{c1k}^{1}\in \Omega ,\;a_{c2k}^{1}\notin \Omega \end{array}$$

If ***a***^1^_c1*k*_ does not belong to *Ω* and ***a***^1^_c2*k*_ belongs to *Ω*, then the objective function *H*_3_ is represented as

(26) $$\begin{array}{l} H_{3}=\underset{\left\{a_{c11},a_{c21},\cdots ,a_{c1n},a_{c2n}\right\}}{\min}\sum_{i=1}^{n-1}{\omega_{c2i}}^{2}\\ \;s.t.\;a_{c1k}^{1}\notin \Omega ,\;a_{c2k}^{1}\in \Omega \end{array}$$

In the actual optimization process, ***a***^3^_c1*k*_ and ***a***^3^_c2*k*_ are defined as the local optimal vectors. If ***a***^1^_c1*k*_ belongs to *Ω*, ***a***^3^_c1*k*_ is the same as ***a***^1^_c1*k*_. If ***a***^1^_c2*k*_ belongs to *Ω*, ***a***^3^_c2*k*_ is the same as ***a***^1^_c2*k*_, otherwise, they are not the same. Therefore, the final tool axis vector ***a***′_ck_ can be expressed as

(27) $${a^{\prime }}_{ck}=a_{c1k}^{2}\cup a_{c1k}^{3}\cup a_{c2k}^{2}\cup a_{c2k}^{3}$$

Based on the above algorithm, all the smoothness orientations can be calculated for the top-to-top contact point, and are all optimal tool axis vectors.

## 4. Simulation and Experimental Verifications

### 4.1. Twin-Tool Orientation Smoothness Planning

**Figure 8:**
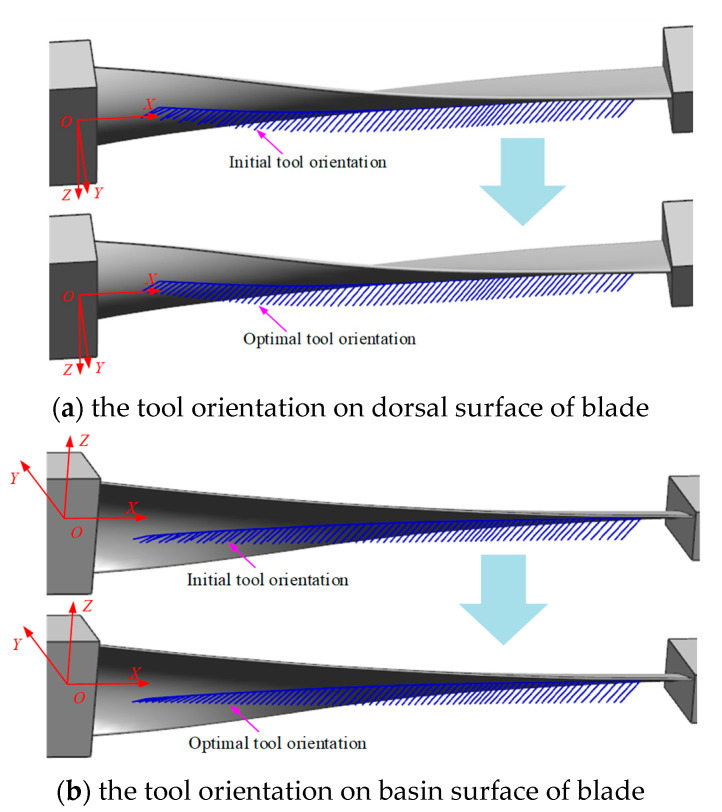
The final twin-tool orientation for pinch milling.

The implementation of the presented twin-tool orientation optimization algorithm was successfully carried out using C++. A turbine blade is used to test the algorithm, as shown in Figure 8, and the length and rotational diameter of the blade are 633 and 165 mm, respectively. It can be seen from this CAD model that the blade body is thin-wall, asymmetric, and torque-shaped; planning the path and tool axis vector for two opposing milling cutters is a complex and challenging task, and, hence, a good test example for the proposed algorithm. The tool path is assumed to be generated from the upper stream procedures.

Two of the same torus-shaped milling cutters with the radius *R* of 16 mm and the corner radius *r* of 6 mm are employed to synchronously mill the oppose surface of the blade, respectively. For the opposing cutting contact points and initial twin-tool axis vectors generated during the path planning of blade profiles, there is no interference during the machining process of the blade surface, and it also satisfies the structural constraints of the two cutters layout. However, there is an obvious nonsmooth phenomenon among the tool axis vectors, and the tool axis vectors are not in a unified sequence.

Using this optimization method, the feasible domain of the two cutters axis vectors are calculated at each cutting contact point, and the tool posture curve is constructed; then, the optimal twin-tool axis vectors are planned through global and local algorithms. The results are shown in Figure 8. By comparing and analyzing the optimization results, it is found that the cutting contact point positions on the path remain unchanged, and the cutting parameters before and after optimization are macroscopically consistent. However, the optimized tool axis vector has visually visible smoothness and changes uniformly along the path, while avoiding interference during the machining process.

**Figure 9:**
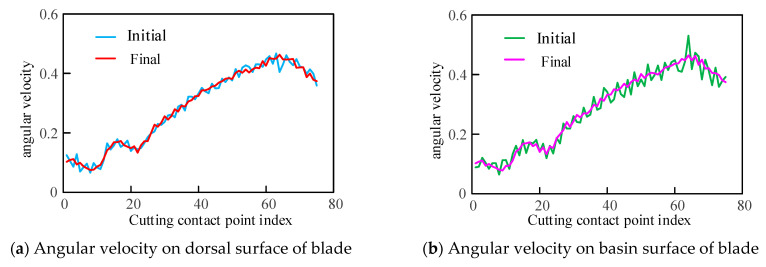
Comparison of angular velocity by initial and finial tool orientation.

The average angular velocities between the initial and optimal tool axis vectors were calculated separately, and the results are shown in Figure 9. The variations in angular velocities are not entirely consistent on both surfaces of the blade, but the significant angle variations between the initial tool axis vectors have been corrected, i.e., the maximum value of the initial tool orientation on the basin surface is 0.5302, and the final is 0.4646. Moreover, the amplitude of variation in average angular velocity after optimization is relatively reduced, and the variations of adjacent tool axes are decreased. It can be seen that the algorithm given obtains continuously changing tool axis vectors with relatively uniform amplitudes of variation, and the planning twin-tool orientation is nearly smoothness.

### 4.2. Pinch Milling Experimental

**Figure 10:**
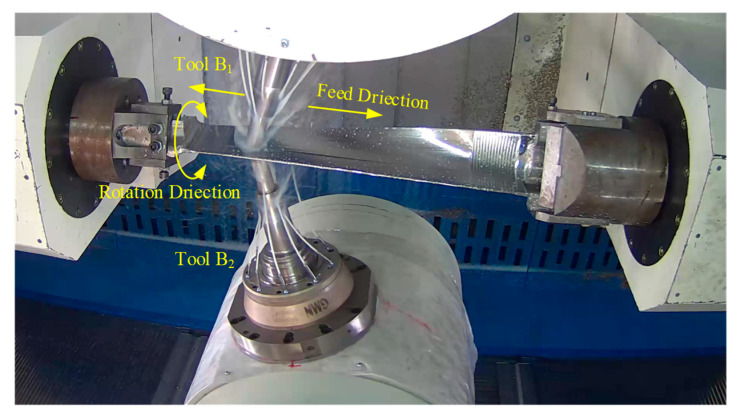
Pinch milling experiment of blade materials.

The nine-axis machine tool for pinch milling was used to validate the proposed tool orientation optimization algorithm. The two cutters are arranged symmetrically with respect to the workpiece rotation, which are both the same torus-shaped milling cutters. Any cutter has an independent rotational speed, which is set to 2200. The material of the blade is 1Cr12Ni2W1Mo1V, which is a difficult-to-machine material. The material of the two cutters is hard alloy. Using the planned optimal tool axis vector, the pinch milling process is implemented for the blade, as shown in Figure 10. It is shown that the twin-tool orientation continuously and uniformly varies along the tool path, the milling process is smoothest, the cutting speed is increased by 46% for the initial process, the noise during the cutting process is noticeably reduced, and cutting stability is achieved along the entire path. The machined surface is smooth and flawless, with no visible interference marks, which is feels delicate and smooth to the touch with low roughness. Meanwhile, the product’s size and shape closely match the specialized inspection mold, and the machining accuracy fully meets the preset requirements. Therefore, the algorithm is very beneficial for ensuring good machining quality of the blade surface. Additionally, it can better leverage the performance of the machine tool to enhance processing efficiency.

## 5. Conclusions

In this research, the twin-tool orientation smoothness planning method is proposed for thin-wall blade with pinch milling. Based on the two cutters structural layout, we clarify the coupling relationship of twin-tool posture, and a calculation method of the feasible region is defined considering multiple constraints. Then, the tool posture curve is constructed for tool orientation, and the global and local optimal planning algorithms are studied within the feasible domain. Furthermore, taking the tool axis vector on the tool posture curve as the optimal value and aiming for the minimum average angular velocity change, the twin-tool synchronization smoothing planning method is proposed for pinch milling.

The optimal twin-tool axis vector are successfully planned for a typical turbine blade using the proposed method, and are nearly smooth, and the collision-free requirement is guaranteed. By simulation and verification experiments, it is shown that the tool axis vectors of two cutters change uniformly along the path, and the pinch milling processing is very smooth. Therefore, efficient and high-precision processing of pinch milling can be achieved, ultimately improving the machining accuracy of the blade with difficult-to-machine materials.

## Data Availability

The data will be provided as required.
